# Peculiar Idiosyncrasy—Treatment Wanted

**Published:** 1892-04

**Authors:** J. L. Bass

**Affiliations:** Brewton, Ala.


### PECULIAR IDIOSYNCRASY.—TREATMENT WANTED.

In bringing this case before the profession, I do so trusting that I may receive assistance from some of the many readers of this journal. The case first came under my observation a year ago. A widow, about thirty years old, general health good, but of rather a nervous temperament, is the subject of a peculiar idiosyncrasy; to inhale the smoke from a pipe immediately causes convulsions, and of a very alarming type. They usually come on about five minutes after the smoke is inhaled, and last about two or three minutes, then pass off to return again after five minutes; continuing in this way from eight to twelve hours, leaving her in a very nervous, weak state and badly nauseated; nausea probably caused by the drugs used. She is usually confined to bed two weeks or longer, and regains her normal condition very slowly.

The muscles of respiration seems to be the only parts affected; she complains of a drawing or tightening sensation about the lungs, as "though the life was being pressed out of them." For thirty seconds before the convulsions come on she gets so restless and nervous it is necessary to hold her in the bed, while she clutches at her breast, screaming with every breath till the convulsion appears and deprives her of consciousness.

The heart's action is good; breathing always irregular and alarming. Various remedies have been used but seem to do little good. The last time I attended her I gave a hypodermic of apomorphia, which seemed to do more good than anything I have tried. Morphine has a bad effect, consequently I avoid using it as much as possible. Chloroform and nitrite of amyl were used for the relief of convulsions. The various nerve sedatives seemed to do but little, if any, good. She has been treated at various times by four or five different physicians, but no one has as yet found a remedy for this peculiar idiosyncrasy.

The lady is a clerk in a dry goods store, and is exposed to this danger at all times, for in every instance when she inhales the smoke from a pipe convulsions is the inevitable result. Cannot some brother assist me in this case? I will be glad to answer any questions, either through this journal or by private letter.

J. L. BASS, M. D.

*Brewton, Ala., March,* 1892.

